# PD83 Medication Preferences Of Rural Patients With Chronic Disease: A Discrete Choice Experiment In An Eastern Province Of China

**DOI:** 10.1017/S0266462324003349

**Published:** 2025-01-07

**Authors:** Xiaona Li, Min Gao, Jiaxian Shao, Yuncong Yu, Wenqiang Yin, Zhongming Chen

## Abstract

**Introduction:**

With the aging population, chronic diseases have become a serious threat to public health in China. Adhering to the doctor’s advice is an effective strategy for controlling chronic diseases, and the preferences of patients with chronic disease has an important impact on compliance with medication. However, there is insufficient research exploring this aspect.

**Methods:**

In this study patients with chronic disease were selected by stratified random sampling to participate in a survey carried out in three cities of a province in eastern China. The discrete choice experiment used a questionnaire of D-efficiency experimental design to measure the medication choices of patients with chronic disease. The main attributes included drug price, onset of action, adverse reactions, traditional Chinese or Western medicine, domestic drug, and reimbursed by medical insurance. The data were analyzed using a mixed logit model.

**Results:**

A total of 1,062 valid questionnaires were received. The 1,045 questionnaires that passed the consistency test covered three prefecture-level cities, nine counties, and 216 villages. All drug attributes were statistically significant for selection preferences. The preference of patients in rural areas with chronic disease was “quick onset of action” (β=2.491), “Western medicine” (β=0. 826), “medical insurance” (β=0.556), “domestic drugs” (β=0.286), and “very few adverse reactions” (β=0.170). “Drug price” also had an impact on medication preferences among patients in rural areas with chronic disease (β=−0.013).

**Conclusions:**

Onset of action is the attribute of medications that is of most concern for patients in rural areas with chronic disease. Subgroup analysis showed that these patients were predominantly female, had a primary school education or lower, were younger than 69 years, were unemployed, and had an annual income between CNY10,000 (USD1,396.78) and CNY50,000 (USD6,983.92). They were willing to pay more for drugs with a quick onset of action, Western medicines, and drugs with reimbursed by medical insurance.

